# Supplementary material for: One Advantage of Being Polyploid: Prokaryotes of Various Phylogenetic Groups Can Grow in the Absence of an Environmental Phosphate Source at the Expense of Their High Genome Copy Numbers
Source: Microorganisms. 2023 Sep 9;11(9):2267. doi: 10.3390/microorganisms11092267 (PMC10536925; doi:10.3390/microorganisms11092267)
Supplement: Supplementary file 1 [file microorganisms-11-02267-s001.zip › microorganisms-2569988-supplementary.pdf]

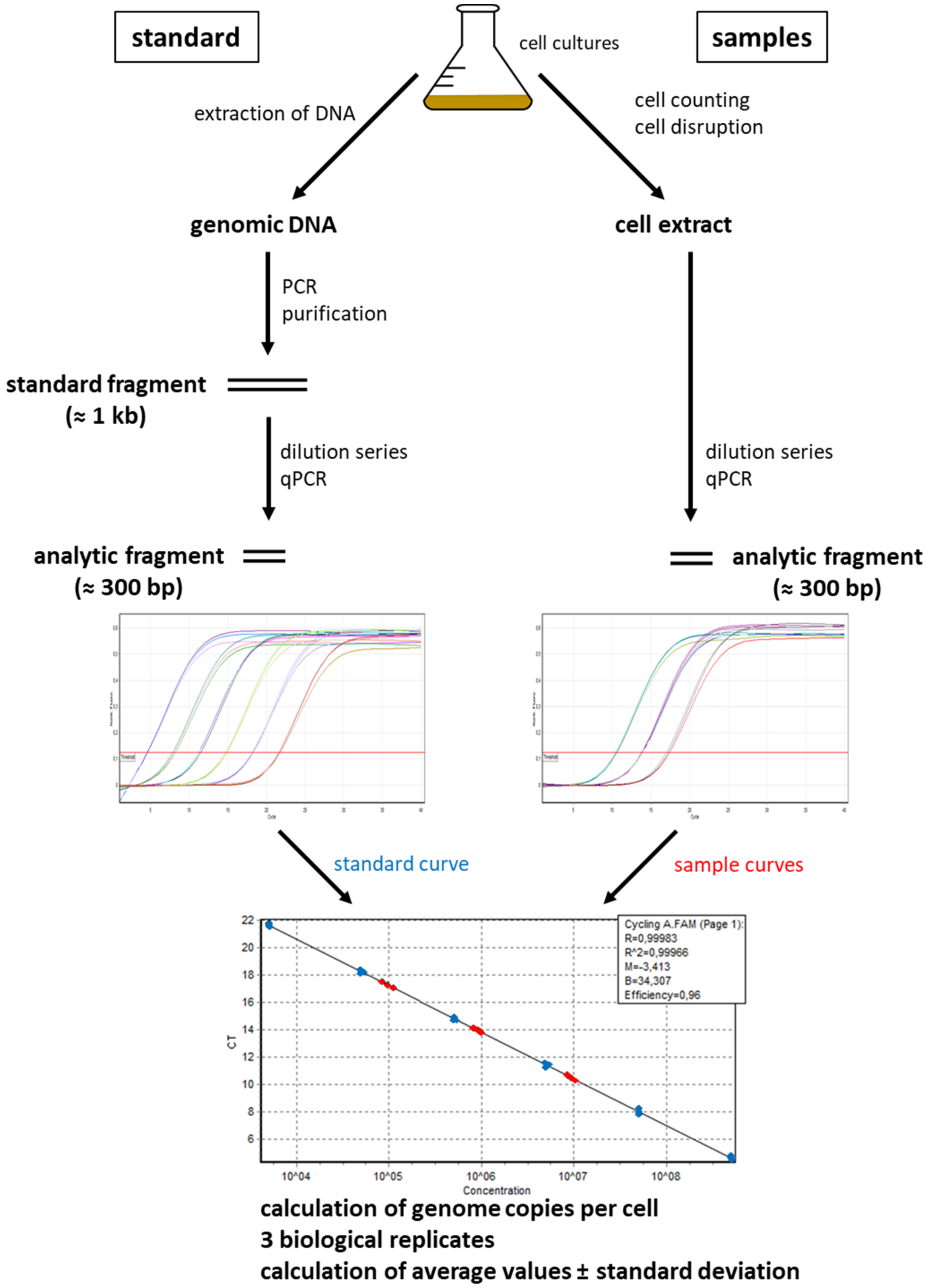

Supplementary Figure S1: Schematic overview of the real-time PCR method for quantification of chromosome copy numbers per cell (taken from Fuchino et al. (see below) with modifications).

Reference:
Fuchino, K.;Wasser, D.; Soppa, J. Genome Copy Number Quantification Revealed That the Ethanologenic α-Proteobacterium *Zymomonas mobilis* Is Polyploid. Front. Microbiol. **2021**, 12, 705895.

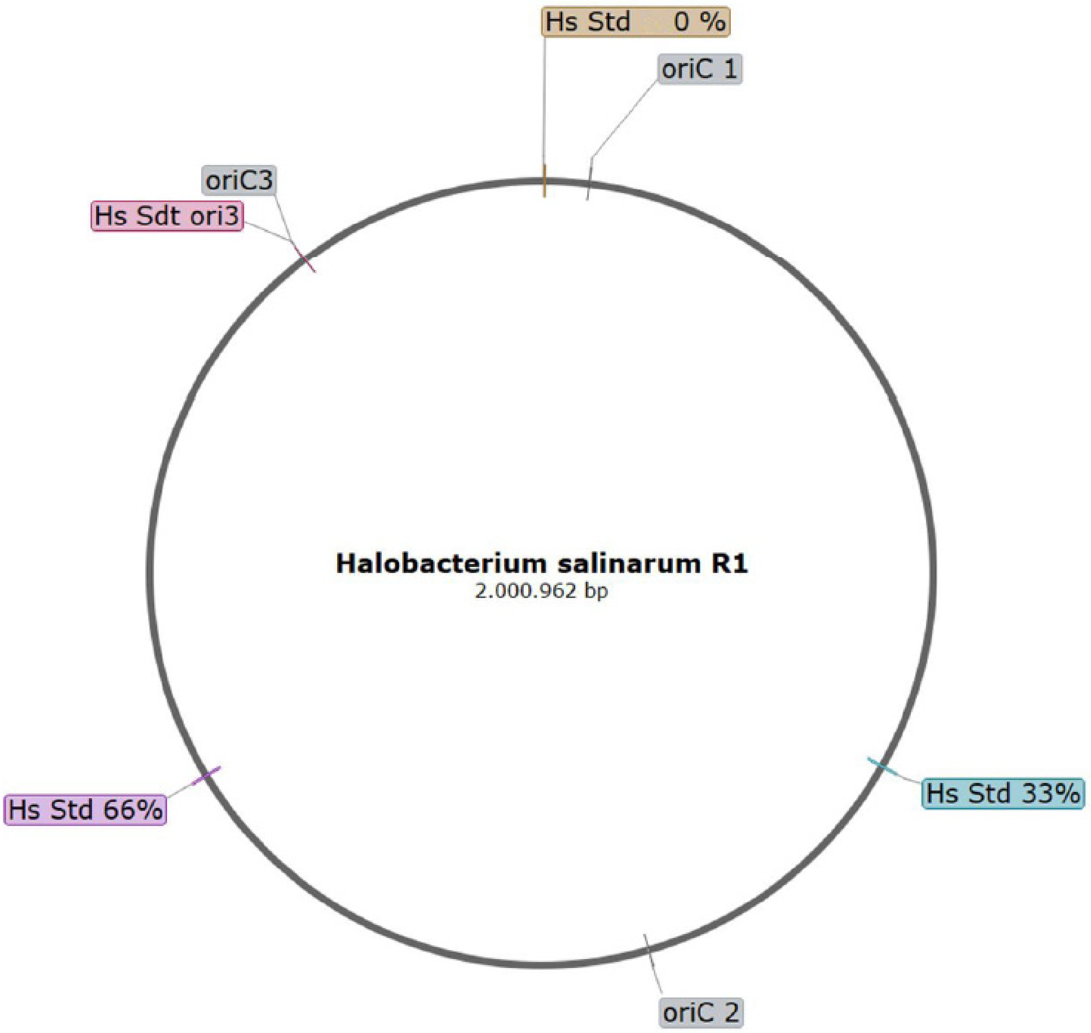

Supplementary Figure S2: Map of the main chromosome of Halobacterium salinarum and locations of all standard fragments. The marked chromosomal locations were utilized for amplifying fourdistinct standard fragments, namely Hs Std ori3, Hs Std 0%, Hs Std 33%, and Hs Std 66%. These standard fragments exhibit varying distances to different origins of replications.

Supplementary Table S1: List of primers with sequences and application.

Name	Sequence (5’ -> 3’)	Application
SB 005 ST 1kb	CCGTTGCGCTCGATTTCGAC	
SB 006 ST 1kb	ACGGCCAGCAAGGCCATCAG	
Hs 33 SF for	GTGAGCTGGCGTTCCTGAACTCC	
Hs 33 SF rev	GCAGCGGCTTCATGTGCCGATAG	standard fragments of
Hs 66 SF for	CGAAGAGCTGCCCGAGGATATCAC	*H. salinarum*
Hs 66 SF rev	ATGCGCACCGCACCGAATAC	
Hs 100 SF for	CGCAGGCGCAGGTCGAACGAATTG	
Hs 100 SF rev	ACGCCAGCCAGCGGGCTAATGTG	
qPCR SB 001 ST	CCACCCGCCAGCCAAGATCAGCGCCCGAAC	
qPCR SB 002 ST	GCAGGCACGATCACAGCAACCCGATACCAG	
Hs 33 A1 for	GAACTCGACGGCGAGAACCTCACC	
Hs 33 A1 rev	GCGTGAGCTCCTCGGCTTTCTTGG	analysis fragments of
Hs 66 A1 for	GCTGGTCGGGTCGAACGTTG	*H. salinarum*
Hs 66 A1 rev	ATGCGCACCGCACCGAATAC	
Hs 100 A1 for	CGACGGCAGTCTGTACCGGATTCG	
Hs 100 A1 rev	TCACGCCCTCACGGGCATCTGAAG	
oriF1	TTCGTGATCGGCCATTGGTTTC	standard fragments of
oriR2	CAATGACCATTTCGGCCACCTG	*Z. mobilis*
oriqF3	ATTACGCAAGCAAGACCTCC	analysis fragments of
oriqR2	ACGGACATTATCGGGATTGTCG	*Z. mobilis*
Av SF1 ori for	CCGGTGGGCACCAGTAGTAAC	
Av SF1 ori rev	CAGGCCGCTGGCAGAGAAAG	standard fragments of
Av SF1 ter for	GAACATGGGACAGGCGCAAAG	*A. vinelandii*
Av SF1 ter rev	CCGGCATGAGGGAATGTTTGG	
Av A1 ori for	GCCTCAGGGCTGAAGCTTTCTTG	
Av A1 ori rev	GCTACGAGGACGGTAGCGAGAATC	analysis fragments of
Av A1 ter for	TCATGCCCGCGACAACAAAC	*A. vinelandii*
Av A1 ter rev	GTACGACGGACATACTGGCAGAC	
Ec SF ori for	GCGGTGAGCAAGGTATTAAG	standard fragments of
Ec SF ori rev	CCGAAATAGCGGGTGTTATG	*E. coli*
Ec A1 ori for	CCATAATGAAGTGGCGTCCTTTCG	analysis fragments of
Ec A1 ori rev	TATCGCCGTAGACAACTCCAATCG	*E. coli*

Supplementary Table S2: Overview of the number of phosphate molecules that are sequestered in the genome of one cell prior to the growth in the absence of an external phosphate source, the number of phosphate molecules in the genomes of the descendants of one cell after the growth in the absence of phosphate, and the difference.

Species	No. P_i mol. in 1 Cell Prior to Starvation	No. P_i mol. in Descendants after Starvation	No. P_i mol. Missing after Starvation	Fraction P_i Missing after Starvation	Reference
Z. mobilis	6.25×10^7	6.08×10^7	0.17×10^7	2.7%	This study
A. vinelandii					
Gluc./Mann.	2.22×10^8	2.44×10^8	-0.22×10^8	−9.9%	This study
Sucrose	3.38×10^8	3.03×10^8	0.35×10^8	10.4%	This study
H. salinarum	1.30×10^8	1.17×10^8	0.13×10^8	10.0%	This study
H. volcanii	2.2×10^8	1.5×10^8	0.7×10^8	31.8%	[52]
